# A paratransgenic strategy to block transmission of *Xylella fastidiosa* from the glassy-winged sharpshooter *Homalodisca vitripennis*

**DOI:** 10.1186/s12896-018-0460-z

**Published:** 2018-08-22

**Authors:** Arinder K. Arora, Kendra N. Pesko, Verónica Quintero-Hernández, Lourival D. Possani, Thomas A. Miller, Ravi V. Durvasula

**Affiliations:** 1 Department of Biology, University of New Mexico, Albuquerque, NM-87131 USA; 2 Department of Entomology, Cornell University, Ithaca, NY-48153 USA; 3 Molecular Biology, Scientific Laboratory Division, New Mexico Dept. of Health, Albuquerque, NM-87102 USA; 4 Departamento de Medicina Molecular, Instituto de Biotecnologia, Universidad Nacional Autonoma de Mexico, Av. Universidad, 2001, Colonia Chamilpa, 62210 Cuernavaca, Morelos Mexico; 5 CONACYT-Laboratorio de Ecología Molecular Microbiana, Centro de Investigaciones en Ciencias Microbiológicas-Instituto de Ciencias, Benemérita Universidad Autónoma de Puebla, Ciudad Universitaria, Col. San Manuel, C.P. 72570 Puebla, Puebla Mexico; 6 Department of Entomology, University of California, Riverside, CA-92521 USA; 7 Present Address: Department of Medicine, Loyola University Stritch School of Medicine, Maywood, IL-60153 USA

**Keywords:** *Xylella fastidiosa*, *Pantoea agglomerans*, *Homalodisca vitripennis*, Paratransgenesis

## Abstract

**Background:**

Arthropod-borne diseases remain a leading cause of human morbidity and mortality and exact an enormous toll on global agriculture. The practice of insecticide-based control is fraught with issues of excessive cost, human and environmental toxicity, unwanted impact on beneficial insects and selection of resistant insects. Efforts to modulate insects to eliminate pathogen transmission have gained some traction and remain future options for disease control.

**Results:**

Here, we report a paratransgenic strategy that targets transmission of *Xylella fastidiosa*, a leading bacterial pathogen of agriculture, by the Glassy-Winged Sharpshooter (GWSS), *Homalodisca vitripennis*. Earlier, we identified *Pantoea agglomerans*, a bacterial symbiont of the GWSS as the paratransgenic control agent. We genetically engineered *P. agglomerans* to express two antimicrobial peptides (AMP)-melittin and scorpine-like molecule (SLM). Melittin and SLM were chosen as the effector molecules based on in vitro studies, which showed that both molecules have anti-*Xylella* activity at concentrations that did not kill *P. agglomerans*. Using these AMP-expressing strains of *P. agglomerans*, we demonstrated disruption of pathogen transmission from insects to grape plants below detectable levels.

**Conclusion:**

This is the first report of halting pathogen transmission from paratransgenically modified insects. It is also the first demonstration of paratransgenic control in an agriculturally important insect vector.

**Electronic supplementary material:**

The online version of this article (10.1186/s12896-018-0460-z) contains supplementary material, which is available to authorized users.

## Background

Despite advances in public health, arthropod vectors continue to exact a toll, either directly through transmission of human pathogens or indirectly by transmitting pathogens to animals and agricultural crops [1]. Plant diseases caused by pathogens that are transmitted by insects such as leafhoppers, planthoppers, aphids, whiteflies and thrips have profound implications on food security [2–4]. The vector borne diseases are managed mainly by controlling insect populations using insecticides. The side effects of chemical pesticides, including secondary pest outbreaks and selection for insect resistance, have confounded efforts to control these diseases and underscore the need to develop new approaches to pathogen control [5]. Paratransgenesis, the modification of symbiotic microorganisms associated with insects, has been developed for several vectors of human pathogens such as triatomine bugs, tsetse flies, sandflies and mosquitoes (i.e., [6–9]). This strategy relies on delivery of anti-pathogen molecules within the insect vector via engineered symbiotic bacteria to make the insect incompetent to carry and transmit the pathogen [6]. Several models of paratransgenic insects have been developed but none to date has been validated as a method to block transmission of a pathogen and prevent disease in a target host. Here, we report the paratransgenic manipulation of an agricultural pest, *Homalodisca vitripennis* (the Glassy-Winged Sharpshooter), to block transmission of the bacterial pathogen, *Xylella fastidiosa*, to grape plants.

*X. fastidiosa* is currently a leading agricultural pathogen globally, as the causative agent of Pierce’s disease (PD) of grapevines, citrus variegated chlorosis (CVC) of citrus crops and olive quick decline of olive trees [10–12]. Xylem-feeding sharpshooters and spittlebugs are the known vectors of *X. fastidiosa* [10, 13]. *H. vitripennis* commonly known as the Glassy-Winged Sharpshooter (GWSS) due to its long-range mobility and high fecundity, is the most important vector in California [14]. We recently identified *Pantoea agglomerans* as a symbiotic bacterium of *H. vitripennis* and, using an EPA-approved non-pathogenic variant of *Pantoea*, reported both paratransgenic manipulation and a field-applicable strategy to target GWSS with engineered bacteria [15]. Using this platform, we have engineered lines of *P. agglomerans* that secrete antimicrobial peptides (AMP) that kill *X. fastidiosa* and report here, for the first time, a pathogen-refractory *H. vitripennis* that is unable to infect target plants.

## Results

### Selection of melittin and scorpine-like molecules (SLM) as effector molecules

Melittin, a 26 amino acid-long peptide having an alpha- helix structure, is found in honeybee venom and kills cells through pore formation or by inducing apoptosis [16]. SLM (dbEST accession: JZ818337) is an AMP found in the venom gland transcriptome of the scorpion *Vaejovis mexicanus* [17]. SLM is a 77 amino acid-long peptide and its amino-terminal region is similar to peptides of the cecropin family. I-TASSER predicted that SLM is composed of three coil-helix structures (Additional file 1: Figure S1) [18, 19].

**Fig. 1:**
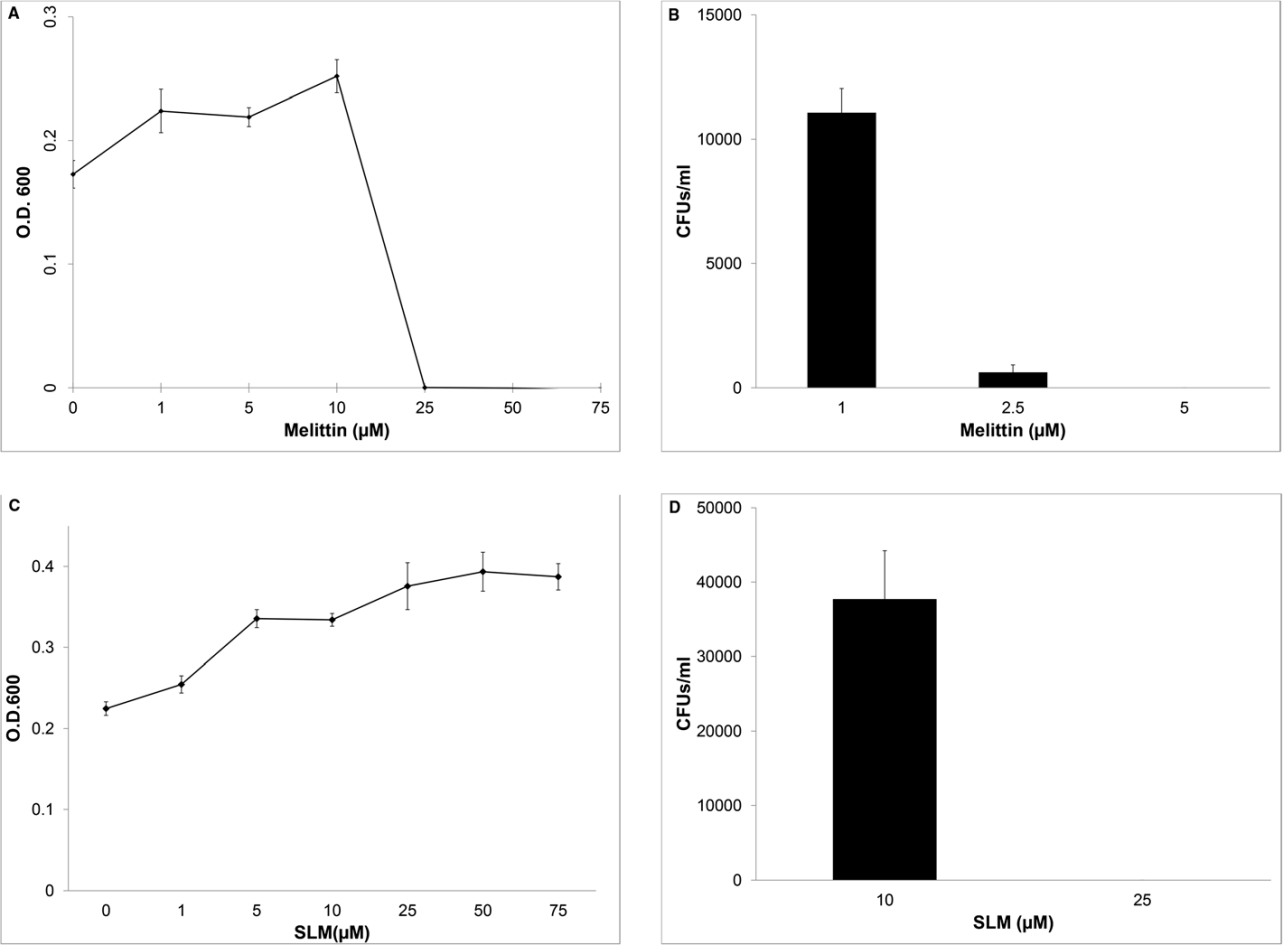
Toxicity of melittin and SLM against *P.agglomerans* and *X. fastidiosa*. 10^5^–10^6^ CFUs of *P. agglomerans* and *X. fastidiosa* were treated with each AMP. O.D. 600 was measured 24 h after treatment of *P. agglomerans* with each AMP. Given the slow growth rate of *X. fastidiosa,* this organism was cultured 24 h after treatment with each AMP and CFUs were counted. *P. agglomerans* O.D.600 after treatment with - (**a**) melittin, **c** SLM; *X. fastidiosa* CFUs counts after treating with - (**b**) melittin, **d** SLM. Both melittin and SLM exerted greater toxicity toward *X*. *fastidiosa* than *P*. *agglomerans*. All values in each graph are combined results from two independent experiments

We tested activity of both peptides against *X. fastidiosa* as well as *P. agglomerans*. Melittin killed *X. fastidiosa* at a concentration of 5 μM, which was 20% of the concentration needed to kill *P. agglomerans* (25 μM) (Fig. 1a and b).

Similarly, SLM killed *X. fastidiosa* at a concentration of 25 μM; it had no effect on *P. agglomerans* even at a concentration of 75 μM (Fig. 1c and d). The selective toxicity of these molecules to *X. fastidiosa* renders them ideal effectors for paratransgenic manipulation of *H. vitripennis*.

### Generation of AMP-expressing *P. agglomerans* strains

It is imperative that melittin and SLM interact with *X. fastidiosa* directly to kill it. To achieve this, *P. agglomerans* should be transformed in a way that the molecules are excreted rather than contained within the bacterial cytoplasm. An *Escherichia coli* hemolysin secretion system that has earlier been used to secrete active proteins into the outside environment of Gram-negative bacteria, was used to genetically engineer *P. agglomerans* to accomplish the goal of AMP secretion [9, 20]. The *E. coli* hemolysin secretion system has two components: HlyA secretion signal and two pore forming proteins, HlyB and HlyD. Peptides with HlyA secretion signal at the carboxyl end are recognized by the pores formed by HlyB and HlyD and are secreted out of the cytoplasm. We introduced genes encoding melittin or SLM in the plasmid, pEHLYA2-SD at the 5′ end of the E-tag, which was in-frame with the HlyA secretion signal (Additional file 2: Figure S2b). Once the AMP genes were cloned into the pEHLYA2-SD plasmid, *P. agglomerans* were transformed with pVDL9.3, a plasmid with HlyB and HlyD genes, and pEHLYA2-SD or pEHLYA2-SD-Mel or pEHLYA2-SD-SLM (See "Methods" for details).

**Fig. 2:**
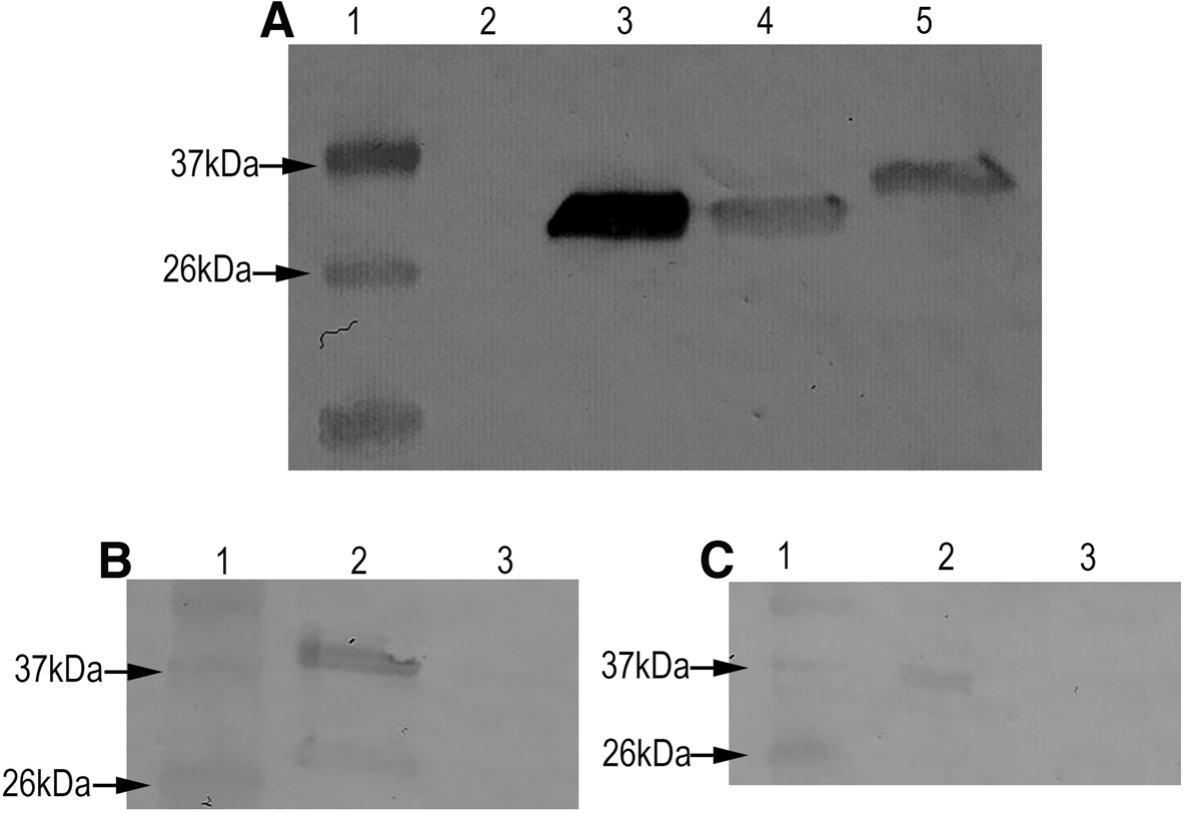
**a** Western blot showing secretion and accumulation of melittin and SLM conjugated to HlyA secretion signal by transformed *P. agglomerans* lines in spent media. Spent media from transformed *P. agglomerans* lines were concentrated using Micron 10 kDa filters. Concentrated spent medium was tested using an anti-E-tag antibody. Lane 1: ladder; lane 2: Wild type *P. agglomerans*; lane 3: HlyA secretion signal only; lane 4: melittin conjugated to HlyA secretion signal; lane 5: SLM conjugated to HlyA secretion signal. **b**, **c** Western blots showing secretion and accumulation of melittin and SLM conjugated to HlyA secretion signal by transformed *P. agglomerans* lines in the GWSS gut. Extracts from homogenized GWSSs were tested for presence of AMPs using an anti-E-tag antibody. **b** Lane 1: ladder; lane 2: GWSS fed on *P. agglomerans* expressing melittin conjugated to HlyA secretion signal; lane 3: GWSS fed on wild type *P. agglomerans* (**c**) Lane 1: ladder; lane 2: GWSS fed on *P. agglomerans* expressing SLM; lane 3: GWSS fed on wild type *P. agglomerans*. Five insects were tested individually for accumulation of SLM and melittin, and two insects were found positive for presence of both AMPs

The spent medium from *P. agglomerans* culture was tested for AMP production via Western blot using anti-E tag antibodies, which demonstrated accumulation of melittin conjugated with HlyA secretion signal (~ 29 kDa), SLM conjugated with HlyA secretion signal (~ 34 kDa) and HlyA secretion signal peptide alone (~ 26 kDa) (Fig. 2a). We also confirmed melittin expression using an anti-melittin bleed, which bound to melittin conjugated to HlyA secretion signal (~ 29 kDa) as well as to synthetic melittin (~ 3 kDa) (Additional file 3: Figure S3a).

### Blocking transmission of *X*. *fastidiosa* from *H. vitripennis*

**Fig. 3:**
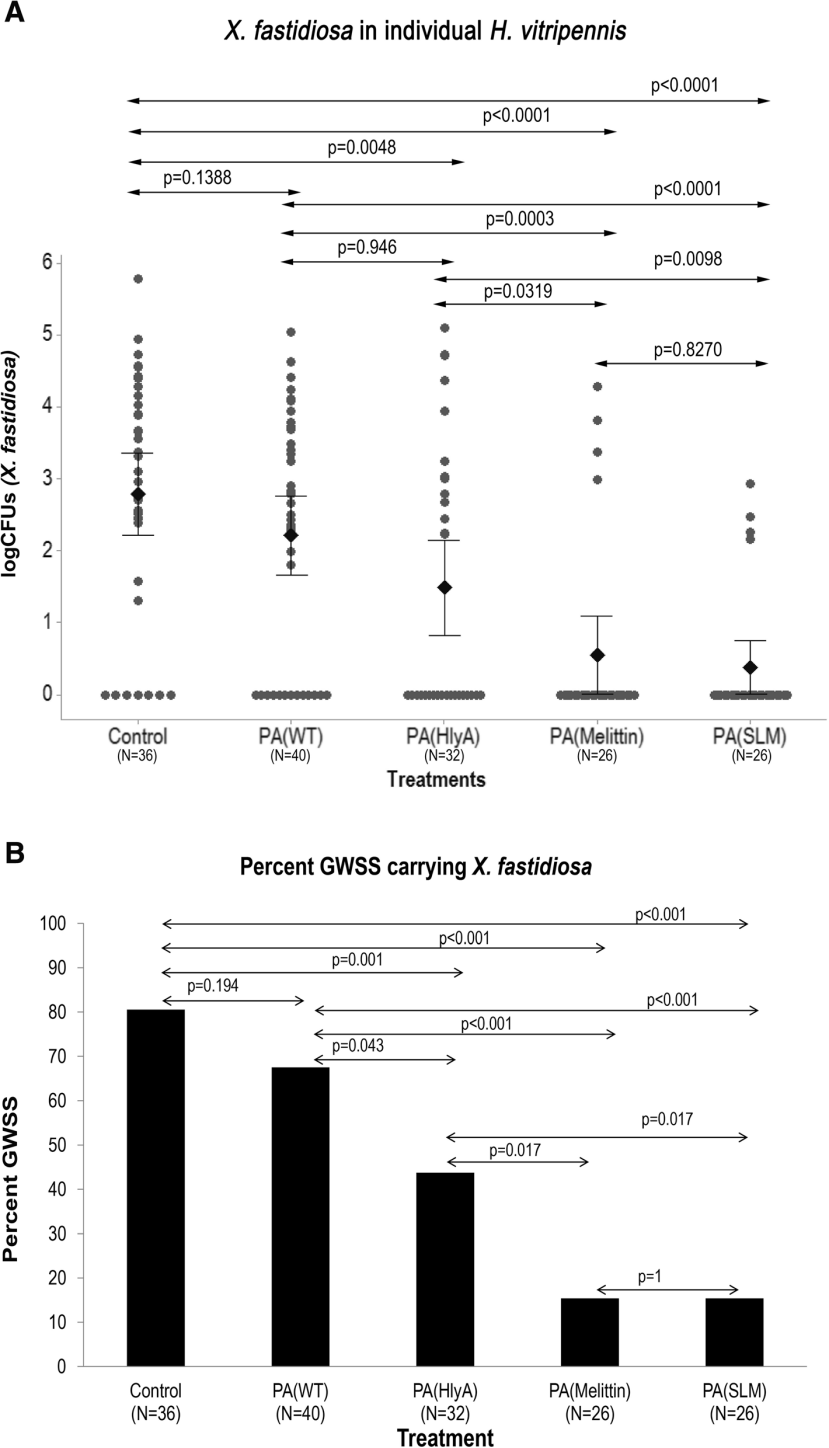
Graphs showing a decrease in *X. fastidiosa* acquisition by paratransgenic GWSSs. *P. agglomerans* was painted on grape stems after mixing with guar gum. PA(WT) - wild type *P. agglomerans*; PA(HlyA) - *P. agglomerans* expressing HlyA secretion signal only; PA(Melittin) - *P. agglomerans* expressing melittin conjugated to HlyA; PA(SLM) - *P. agglomerans* expressing SLM conjugated to HlyA. The GWSSs were allowed to feed on *Pantoea*-painted plants for 48 h before putting them in a cage containing *X. fastidiosa-*infected plants for 48 h. Subsequently the GWSSs were collected and two GWSSs were caged per single naive grape plant for 24 h. These GWSSs were surface sterilized and *X. fastidiosa* presence was assayed using rt-PCR. **a** *X. fastidiosa* CFUs per insect head; **b** Percent of GWSSs carrying *X. fastidiosa*. These are pooled results from two independent experiments

Results from two independent experiments were pooled after confirming that the experiments did not affect the outcome using a generalized linear mixed model. GWSS that harbored AMP-producing *P. agglomerans* were refractory to *X. fastidiosa* acquisition; insects that carried melittin- or SLM-secreting *P. agglomerans*, on an average, had *X. fastidiosa* burden that was 4.3% and 0.2%, respectively, of the pathogen burden in control insects (*p* < 0.001) (Fig. 3a). Furthermore, the number of paratransgenic GWSS that carried *X. fastidiosa* in their foregut also decreased, significantly: 80.6% of control sharpshooters acquired *X. fastidiosa*, while only 15.4% of GWSS harboring melittin- and SLM-secreting *P. agglomerans* were found to carry *X. fastidiosa* in their foregut (*p* < 0.001) (Fig. 3b). Secretion of the HlyA signal peptide alone by *P. agglomerans* in GWSS also decreased acquisition of *X. fastidiosa*; 43.8% GWSS in this group acquired *X. fastidiosa* in their foregut. This is not a surprising result, since the HlyA peptide does disrupt the membrane of Gram negative bacteria and an anti-*Xylella* effect is likely.

**Fig. 4:**
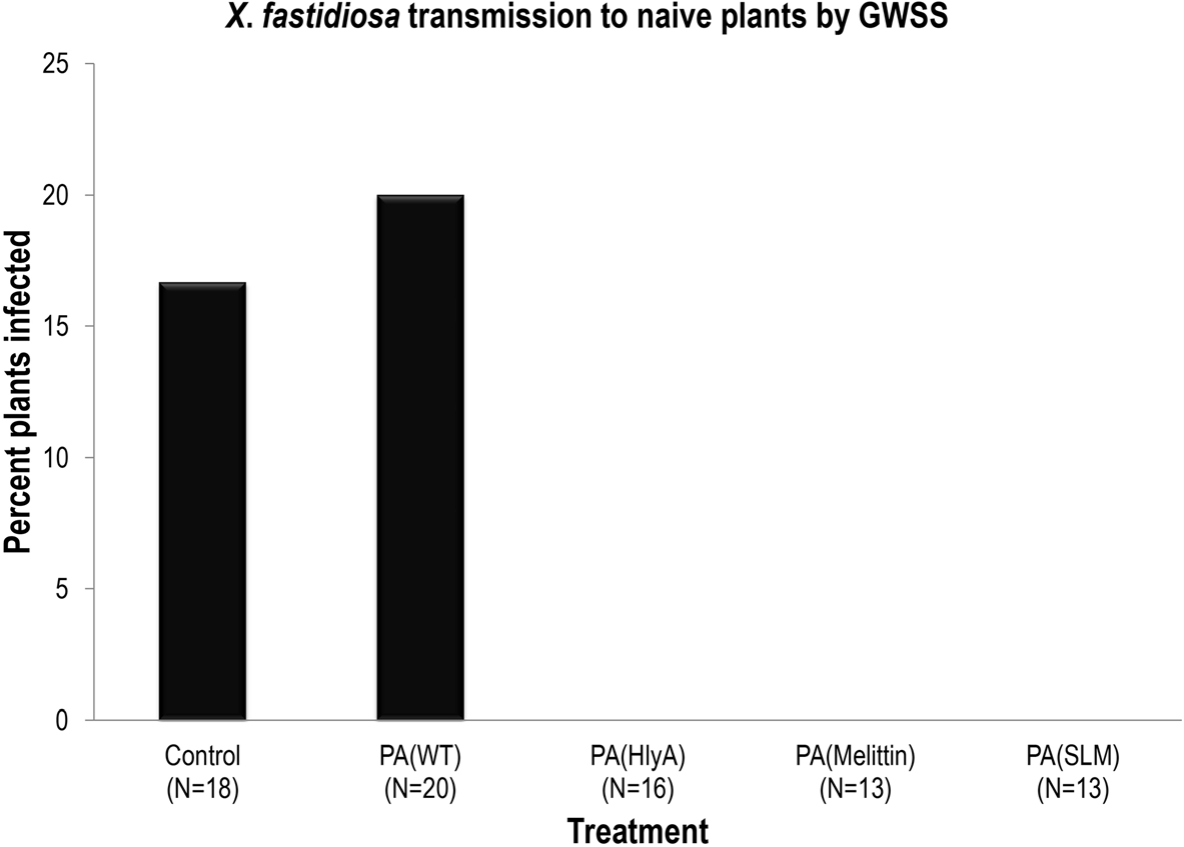
Decrease in *X. fastidiosa* transmission to grape plants by paratransgenic GWSSs. *P. agglomerans* were painted on grape stems after mixing with guar gum. PA(WT) - wild type *P. agglomerans*; PA(HlyA) - *P. agglomerans* expressing HlyA secretion signal only; PA(Melittin) - *P. agglomerans* expressing melittin conjugated to HlyA; PA(SLM) - *P. agglomerans* expressing SLM conjugated to HlyA. The GWSSs were allowed to acquire *P. agglomerans* from *P. agglomerans*-painted plants for 48 h before an acquisition access period of 48 h on *X. fastidiosa*-infected grape plants. Subsequently the GWSSs were collected and two GWSSs were then confined per naive grape plant. After 24 h of inoculation access, the insects were removed and the plants were kept in a greenhouse for 30 weeks before testing them for presence of *X. fastidiosa* using rt-PCR. GWSSs that acquired *P. agglomerans* expressing HlyA secretion signal, melittin conjugated to HlyA secretion signal and SLM conjugated to HlyA secretion signal did not transmit *X*. *fastidiosa*. These are pooled results from two independent experiments

The paratransgenic GWSS that acquired melittin- and SLM- producing *P. agglomerans* strains prior to acquisition of *X. fastidiosa*, failed to transmit *X. fastidiosa* to the naïve grape plants, indicating decreased acquisition of *X. fastidiosa* by *H. vitripennis* resulted in decreased pathogen transmission to naïve grape plants (Fig. 4). Control GWSS and GWSS carrying wild type *P. agglomerans* transmitted *X. fastidiosa* 16.7% and 20% of the time, respectively. GWSS that carried *P. agglomerans,* which secreted only the HlyA signal protein (and not the AMP molecules) also failed to transmit *X. fastidiosa* to the naïve plants.

### Expression of AMP within *H. vitripennis*

GWSS that fed on AMP-expressing *P. agglomerans* were tested for presence of recombinant AMP molecules to confirm that decrease in *Xylella* transmission to grapevines was a result of AMP activity in the insect gut. Western blot analysis confirmed presence of both melittin and SLM with attached HlyA secretion signals within the insects (Fig. 2b and c). Further, we confirmed presence of melittin using anti-melittin serum (Additional file 3: Figure S3b).

## Discussion

Prior studies with paratransgenic insect vectors demonstrated reduction or elimination of pathogens in the insects [6, 9]. Here, we report a paratransgenic strategy that completely eliminates the detectable transmission of a pathogen from an arthropod to a target plant. Three molecules- the HlyA protein, melittin and SLM- when expressed in the GWSS via engineered *P. agglomerans*, blocked transmission of *X. fastidiosa* to grape plants. Melittin and SLM decreased *Xylella* CFUs in paratransgenic GWSS to levels that should eliminate pathogen transmission even during periods of feeding that exceed the 24 h window used in our experimental model. Additionally, under field conditions, several GWSS may feed on a single plant, unlike our experimental model in which only 2 insects were placed on target plants. Again, the level of elimination of *X. fastidiosa* in the insect achieved with melittin and SLM should block transmission under such real world conditions. HlyA alone did reduce *X. fastidiosa* acquisition by the GWSS and eliminated transmission in our study. Similar results were also observed by Wang et al. [9] in paratransgenic mosquitoes, wherein they observed a 32% decrease in *Plasmodium* prevalence in mosquitoes carrying HlyA secretion signal-expressing *P. agglomerans*, though this reduction was not significant statistically. We believe that the impact of HlyA on *X. fastidiosa* may be more pronounced than the effect on Plasmodia due to greater susceptibility of the bacterial cell membrane. However, the degree of *X. fastidiosa* reduction in the insect that is due to HlyA alone may not prevent transmission of the pathogen under conditions of prolonged feeding under field settings.

Almeida and Purcell [21] reported transmission efficiency of 35.3% after 96 h of acquisition and inoculation access using a model with a single GWSS per plant. However, we used two insects per plant with an acquisition and inoculation access of 48 h and 24 h, respectively, and a transmission of 20% was observed in the control group. Future studies will require varying acquisition and inoculation access times and increased number of insects per plant to simulate vector pressure under field conditions to understand the overall impact of transformed bacteria on acquisition and inoculation efficiency of the insect.

Our lab has also developed single chain antibodies (scFvs) specific to the *X*. *fastidiosa* surface protein, mopB [22]. These antibodies can be expressed in tandem with active AMPs or as antibody:AMP chimeras to increase killing efficacy and further reduce target resistance. We are also working on developing antibodies targeting different membrane proteins and pili present on the surface of *X. fastidiosa.* A theoretical concern exists for evolution of resistance amongst target *X. fastidiosa* populations. These antibodies in combination with other AMPs may slow resistance development.

In our experiments, 10^10^ CFU of *P. agglomerans* were painted on each plant. This is, indeed, a high concentration of bacteria but one that was readily administered using a hand-painted approach. The high bacterial concentrations in this study were intended as proof-of-concept. In future applications, different bacterial concentrations need to be tested to determine the threshold CFU required to break transmission cycles. Antibody-AMP chimeras are known to increase the potency of effector molecules [23] and, perhaps, can be used to make the insect incompetent of acquiring the pathogen at lower CFU’s.

Field collected GWSS have been reported to carry different bacteria within their foregut other than *X. fastidiosa* [24–26] and to our knowledge no adverse effect of these colonizing bacteria on insect health has been reported. For instance, *P. agglomerans* expressing EGFP was able to colonize the GWSS gut without impacting the insect’s health [15]. We also did not observe adverse physiological effects in the GWSS carrying *P. agglomerans* strains, such as decreased feeding or early mortality. This suggests that paratransgenic GWSS could be able to complete their life cycle without a negative selection pressure from recombinant symbiotic bacteria. We anticipate that this will allow persistence of recombinant bacteria and, possibly, spread amongst field populations of GWSS. The balance between persistence of recombinant bacteria, spread within a GWSS population and need for repeated applications of engineered symbionts can be addressed in additional field studies.

The full potential of the paratransgenic control method under field conditions has not yet been realized, largely due to lack of delivery strategies that target arthropods. We have recently developed a strategy based on calcium-alginate microparticles to disseminate genetically modified bacteria in the field. These microparticles not only provide a physical barrier between the bacteria and the outer environment to decrease environmental contamination, but also provide protection against desiccation and UV radiation [15].

Spread of pathogens that cause Pierce’s disease and other vector-borne diseases depends largely on the control of arthropod populations with insecticides. There are reports of development of resistance in many insect vectors including mosquitoes and triatomine bugs against insecticides [27–30]. Paratransgenic control of these diseases is an alternative, which can be employed in the field to decrease transmission. It can also be included in integrated vector management. Paratransgenic control may help to reduce spread of human and plant diseases and may decrease over-reliance on chemical pesticides.

The paratransgenic model for PD control may write a new chapter in the control of diseases caused by pathogens carried by agricultural vectors. Whiteflies, aphids, leafhoppers and thrips transmit deadly pathogens to crop plants ranging from cotton to sugarcane to papaya to rice [31–36]. These insects carry bacterial symbionts that enhance their fitness. Future directions of paratransgenic control for agricultural diseases may employ these symbionts as “Trojan Horses” to block transmission of pathogens.

## Conclusion

We report, for the first time, protection of a target from a vector-borne disease, using paratransgenic control. Furthermore, we report the first potential agricultural application of paratransgenic control and are confident that transgenic symbiotic bacteria can be used individually or as a component of integrated vector management to protect crops from threats such as Pierce’s disease.

## Methods

### The glassy-winged sharpshooters (GWSS) maintenance

The Glassy-Winged Sharpshooters (*H. vitripennis*) were collected from crepe myrtle, *Lagerstroemia* sp. trees planted in parking lot 9 of UC, Riverside. These GWSS were kept on basil plants until they were used. A laboratory-based method for propagation of GWSS is not yet available, necessitating use of field-caught arthropods.

### Bacterial strains, culture conditions

*Escherichia coli* strain XL1-Blue was used to maintain plasmids and for gene cloning. *Pantoea agglomerans* E325, an EPA approved biological control agent, was used to express and deliver different AMP molecules inside the GWSS gut. Both *E. coli* and *P. agglomerans* were grown in Luria Bertani agar or broth. *P. agglomerans* and *E. coli* were cultured on agar plates at 30 °C and at 37 °C, respectively. Broth cultures were grown at the same temperatures in a shaker incubator (200 rpm). Carbenicillin or chloramphenicol was added at a concentration of 100 μg/mL and 35 μg/mL, respectively, when needed. Two plasmids that were used in study pEHLYA2-SD and pVDL9.3 have carbenicillin or chloramphenicol resistance markers, respectively.

*X. fastidiosa* Temecula strain was used in this study and was cultured in PD3 agar at 28 °C or in PD3 broth at 28 °C. The culture was agitated at 175–200 rpm to grow *X. fastidiosa* in broth culture.

### Plant inoculations

*X. fastidiosa* strain Temecula was grown in PD3 medium using the conditions as described previously. The bacteria were harvested in log phase and washed thrice with PBS before resuspending in PBS and brought to an O.D. 600 of 0.25, which is an equivalent of 10^8^ cells/ml. Twenty μL of bacterial suspension was inoculated twice into the vine using a needle. The stem was pricked above the second leaf using the needle and one drop of *X. fastidiosa* suspension (2X10^6^) was placed on the point of inoculation; the negative pressure of xylem internalized the bacterial suspension. The plants were kept for 15 weeks before they were used.

### MIC and MBC of AMPs against *P. agglomerans* and *X. fastidiosa*

*P. agglomerans* was grown in LB broth at 200 rpm in a shaker incubator at 30 °C for 16 h. Afterwards *P. agglomerans* was diluted 1/100 in 3 mL LB broth and grown at 30 °C to mid-log phase. At mid-log phase the bacteria were diluted in LB medium to 10^5^–10^6^ colony forming units/mL (CFUs/mL). Ninety μL of diluted *P. agglomerans* were pipetted into sterilized 0.2 mL PCR tubes and to this 10 μL of 10X test concentration of either melittin or SLM (both synthesized by China Peptides, Shanghai, China) was added. These tubes were incubated at 30 °C and after 16 h of incubation OD 600 was determined to ascertain MIC (minimum inhibitory concentration) of AMPs against *P. agglomerans*.

*X. fastidiosa* strain Temecula was grown in PD3 medium in a shaker incubator at 28 °C and 200 rpm until it reached its log phase. Afterwards the *X. fastidiosa* culture was diluted to a concentration of 10^5^–10^6^ CFUs/mL in PD3 medium. Ninety μL of diluted *X. fastidiosa* were mixed with 10 μL of 10X test concentration either AMP in a sterilized 0.2 mL PCR tube and was incubated at 28 °C in a shaker incubator for 16 h. *X. fastidiosa* is a slow growing bacterium, which makes measuring change in OD 600 of overnight cultures unfeasible. Hence, after treatment with AMPs *X. fastidiosa* was plated on to PD3 agar to determine MBC (minimum bactericidal concentration) of AMPs against *X. fastidiosa*. These plates were incubated at 28 °C for 10 days and CFUs were counted. The toxicity assays were repeated twice with three replicates for each dose in each experiment.

### Plasmid construction

Sense and antisense sequences of the melittin gene with *NheI* and *XmaI* overhang were ordered from IDT (Coralville, Iowa, USA) and were annealed to themselves by lowering the temperature by 1 °C/min from 95 °C to 50 °C.

Scorpine like molecule (SLM), an AMP from *Vaejovis mexicanus* venom*,* gene was amplified from a plasmid (kindly provided by Dr. Lourival D. Possani) using forward primer (ScoHlyAF1.1) CAGCTAGCGGTTGGATAAGCGAG; and reverse Primer (ScoHlyAR1.1) TTTTTTATAGGCACGGGGTATACC. The product was cut using restriction enzymes *NheI* and *SmaI*.

The plasmid pEHLYA2-SD (kindly provided by Dr. Luis A. Fernandez, National Center for Biotechnology, Madrid, Spain) - having the hlyA secretion signal of the *E. coli* hemolysin secretion system and *bla* (*β-lactamase*) gene as marker- was also cut using restriction enzymes *NheI* and *SmaI*. Melittin or SLM genes were ligated into linearized pEHLYA2-SD plasmid (Additional file 2: Figure S2). The in-frame presence of both melittin and SLM genes was confirmed by sequencing. The in frame insertion of melittin or SLM gene resulted in plasmid pEHLYA2-SD-Mel or pEHLYA2-SD-SLM.

### *P. agglomerans* transformation

*P. agglomerans* was cultured in LB broth and grown to an OD600 of 0.6–0.7 (mid-log phase). These cells were centrifuged at 4 °C and 8000 rpm for 10 mins and supernatant was removed. The cells were washed with ice cold autoclaved water. The final cell pellet of competent cells was re-suspended in 1 mL 10% glycerol. Eighty μL of competent cell suspension were aliquoted into microcentrifuge tubes. One μL of pVDL9.3 (chloramphenicol resistance as marker) plasmid was added to 80 μL of competent cells and transferred to an ice cold 1 mm cuvette. These cells were electroporated at 2.0 kV, 25 microF. The cells were then plated onto chloramphenicol-containing LB agar. Sixteen hours after plating the colonies were selected and presence of plasmid was confirmed.

pVDL9.3 plasmid-containing *P. agglomerans* cells were made competent using the above mentioned protocol and were transformed with plasmid pEHLYA2-SD or pEHLYA2-SD-Mel or pEHLYA2-SD-SLM. *P. agglomerans* containing both the plasmids were selected on LB agar containing carbenicillin and chloramphenicol.

### Anti-melittin bleed production and ELISA

The anti-melittin bleeds were produced by GenScript (Piscataway, NJ, USA) using manufacturer’s standard protocol (https://www.genscript.com/custom-rabbit-polyclonal-antibody-service.html). Once the bleeds were received the ELISA was conducted on dilutions of melittin ranging from 30 nM to 10 μM. Hundred μL of each dilution was pipetted into the wells of 96-well plate in triplicate. The plate was sealed using plastic wrap and incubated at 37 °C for 2 h. After the incubation the wells were washed 3 times with 250 μL TBST (tween 20 + tris-buffered saline). After washing, the wells were filled with 250 μL of 2.5% BSA (bovine serum albumin)-TBST and sealed. After 2 h of incubation at room temperature the wells were washed three times with 250 μL TBST before adding 100 μL of anti-melittin bleed (1:5000 dilution in TBST) in each well and sealed. The plate was incubated at room temperature for 2 h. Afterwards the plate was washed with 250 μL TBST 3 times and 250 μL of secondary antibody (goat-anti rabbit,1:5000 dilution in TBST) was added, sealed and covered with aluminum foil. The plate was incubated for 1 h at room temperature. After incubation the plate was washed three times with 250 μL TBST and 100 μL of TMB (3,3′,5,5’-Tetramethylbenzidine) was added and covered with aluminum foil. After 30 min of incubation at room temperature the reaction was stopped by adding 100 μL of 18 M H2SO4 per well and the plate was read at 450 nm. The lowest detection limit of the bleed was 1 μM (Additional file 4: Figure S4).

### Detection of melittin and SLM in spent medium

*P. agglomerans* grown in LB for 16 h was centrifuged at 10000 rpm and the supernatants were collected. The supernatant from each culture was concentrated using 10 kDa NMWL filter (EMD Millpore, Temecula, CA, USA). Twenty μL of concentrated spent medium was mixed with 5 μL of loading dye and run on a 8–16% precast polyacrylamide gel (Biorad, Hercules, Califirnia, USA) at a constant electric potential of 150 V. The proteins were then transferred to a nitrocellulose membrane. The nitrocellulose membrane was first incubated with primary rabbit anti-E-tag antibody (Abcam, Cambridge, MA), which was diluted to 1:1000 in 10% milk-TBST, at room temperature. This membrane was washed 5 times with TBST and incubated with mouse anti-rabbit antibody with AP (alkaline phosphatase) conjugate, which was diluted in milk-TBST to 1:5000. This membrane was washed 5 times with TBST and was developed using NBT (nitro blue tetrazolium) and BCIP (5-bromo-4-chloro-3-indolyl-phosphate).

Presence of melittin in the supernatant was reconfirmed using rabbit anti-melittin bleed using the protocol as mentioned above. Anti-melittin bleeds were generated by injecting five rabbits with synthetic melittin and subsequently testing activity of bleeds harvested from these rabbits against melittin via ELISA.

### *X. fastidiosa* transmission blocking assays

*P. agglomerans* lines were cultured in LB broth for 16 h and cultures were washed twice with PBS. After washing, 10^10^ CFUs of *P*. *agglomerans* lines were suspended in 3 mL PBS. Each suspension was mixed with 20 mL 3% guar gum (*w*/*v*). One mL glycerol and 500 μL India Ink were added to it before this slurry was painted on to grape stems (cv: Chardoney). The plants were kept overnight to let the guar gum dry. These plants were then covered with sleeve cages and field collected GWSS were released on these plants. The sharpshooters were kept on these plants for 48 h before putting them on *X. fastidiosa*-infected plants for another 48 h. After acquisition access of 48 h on *X. fastidiosa*-infected plants, the GWSS were collected and two of these GWSS were confined on naive grape plants for 24 h. The insects were removed after 24 h, surface sterilized and DNA was extracted before running real-time PCR. The inoculated grape plants were kept in the greenhouse for 30 weeks and were tested for *X. fastidiosa* infection via real-time PCR. The experiments were conducted according to the institute guidelines. The experiment was repeated independently and the results were pooled together.

### DNA extraction from the insect head

The GWSS were surface sterilized by washing in 70% ethanol for 2 mins followed by in 10% bleach for 2 mins and rinsed twice in sterilized water for 2 mins. The heads were removed from the sterilized GWSS using a surgical blade. The GWSS heads were then homogenized in 200 μL PBS using a Kontes homogenizer and DNA was extracted using DNeasy Blood and Tissue Kit (Qiagen, Valencia, CA, USA) following manufacturer’s instructions.

### DNA extraction from plant tissues

After 30 weeks of inoculation, stems of 10 cm were cut from the plants. These stems were sterilized by washing in 70% ethanol and 10% bleach for 2 mins each, followed by 2X washing in sterilized water for 2 mins. These stems were put in Adagia bags (Elkhart, IN, USA) and homogenized in 800 μL of lytic buffer (20 mM Tris-Cl pH 8.0, 70 mM sodium EDTA, 2 mM sodium chloride, 20 mM sodium metabisulfite) using mortar and pestle. Two hundred μL of plant tissue suspension in lytic buffer was placed in a 1.5 mL microcentrifuge tube. Each suspension was incubated at 55 °C for 1 h after adding 40 μL of 5% sodium sarkosyl and 1.5 μL of proteinase K. After 1 h of incubation this suspension was centrifuged at 13000 rpm for 15 mins and supernatant was collected. DNA was purified from the supernatant using a GeneClean kit (MP Biomedicals, Santa Ana, CA, USA) following manufacturer’s instructions.

### Real-time PCR

We used ITS-specific primers and probes described in Schaad et al. [37] to run real time-PCR. The 20 μL reaction was performed in 0.1 mL strip tubes containing 10 μL 2X IQ Supermix (Biorad, Hercules, CA), 100 nM forward primer, 200 nM reverse primer, 200 nM Taqman probe with dye, 5.8 μL of PCR-grade water and 2 μL of template DNA. The real-time PCR was performed on the Eppendorf Realplex at 95 °C for 3 mins for enzyme activation followed by denaturation at 95 °C for 15 s, and extension and annealing at 62 °C for 1 min. The PCR was run for 40 cycles.

### Detecting accumulation of AMPs inside the insect body

The Glassy-Winged Sharpshooters were surface sterilized as mentioned above. The whole GWSS were then homogenized in PBS using a Kontes homogenizer. The homogenized solution was then centrifuged at 13,000 rpm for 10 mins and supernatant was used for AMP detection. Twenty μL of supernatant was mixed with 5 μL of reducing marker and was run on precast Mini PROTEAN TGX gels (Biorad, Hercules, Califirnia, USA). Proteins were transferred on to nitrocellulose membranes as mentioned above and accumulation of protein was detected using primary rabbit anti-E-tag antibody as mentioned above.

Accumulation of melittin inside the insect body was confirmed using rabbit anti-melittin serum. The protocol for Western blot is mentioned above.

### Statistical analysis

Chi-square tests for homogeneity were employed to compare number of GWSS carrying *X. fastidiosa* in their foreguts. *X. fastidiosa* CFUs present in GWSS foregut in various treatments were analyzed by Tukey’s test for multiple comparisons after taking log values of CFUs. All values are shown as mean ± S.E. Statistical analyses were performed using Minitab version 17 for windows8. *p* values< 0.05 were considered significant.

## Additional files

Additional file 1: **Figure S1.** The amino acid sequence and depiction of the three-dimensional structure of scorpine like molecule (SLM). (a) SLM sequence showing predicted domains with helix-coil structure. (b) 3-D structure of SLM as depicted by I-TASSER. (DOCX 258 kb)

Additional file 2: **Figure S2.** (a) Assembly and working mechanism of *Escherichia coli* hemolysin secretion system. HlyB and HlyD form pores in the internal membrane of Gram negative bacteria. These pores join pores formed by TolC in the outer membrane and provide a passage to proteins with the HlyA secretion signal. (b) Depiction of cloning of AMP genes in pEHLYA2-SD plasmid. (DOCX 137 kb)

Additional file 3: **Figure S3.** Confirmation of secretion and accumulation of melittin conjugated to HlyA secretion signal by transformed *P. agglomerans* lines in spent media as well as within the sharpshooter gut. (a) Spent media were concentrated and were analyzed using an anti-melittin bleed via Western blot. Lane 1: melittin conjugated to HlyA secretion signal; lane 2: synthetic melittin; lane 3: ladder. (b) A sharpshooter homogenate was analyzed using an anti-melittin bleed. Lane 1: ladder; lane 2: sharpshooter fed on *P. agglomerans* expressing melittin conjugated to HlyA secretion signal; lane 3: sharpshooter fed on wild type *P. agglomerans.* Two glassy-winged sharpshooters were tested for the presence of melittin using an anti-melittin bleed and both were positive. (DOCX 157 kb)

Additional file 4: **Figure S4.** ELISA to determine detection limit of anti-melittin bleed. Different concentrations of synthetic melittin were prepared and detected via ELISA using anti-melittin bleed. These results are pooled results of two independent experiments. (DOCX 57 kb)

## Abbreviations

AMP: Antimicrobial peptide
CFU: Clony forming units
GWSS: Glassy-Winged Sharpshooter
MBC: Minimum bactericidal concentration
MIC: Minimum inhibitory concentration
PD: Pierce’s disease
SLM: Scorpine-like molecule
