# Supplementary material for: A paratransgenic strategy to block transmission of Xylella fastidiosa from the glassy-winged sharpshooter Homalodisca vitripennis
Source: BMC Biotechnol. 2018 Aug 22;18:50. doi: 10.1186/s12896-018-0460-z (PMC6104007; doi:10.1186/s12896-018-0460-z)
Supplement: Supplementary file 1 — Figure S1. The amino acid sequence and depiction of the three-dimensional structure of scorpine like molecule (SLM). (a) SLM sequence showing predicted domains with helix-coil structure. (b) 3-D structure of SLM as depicted by I-TASSER. (DOCX 258 kb) [file 12896_2018_460_MOESM1_ESM.docx]

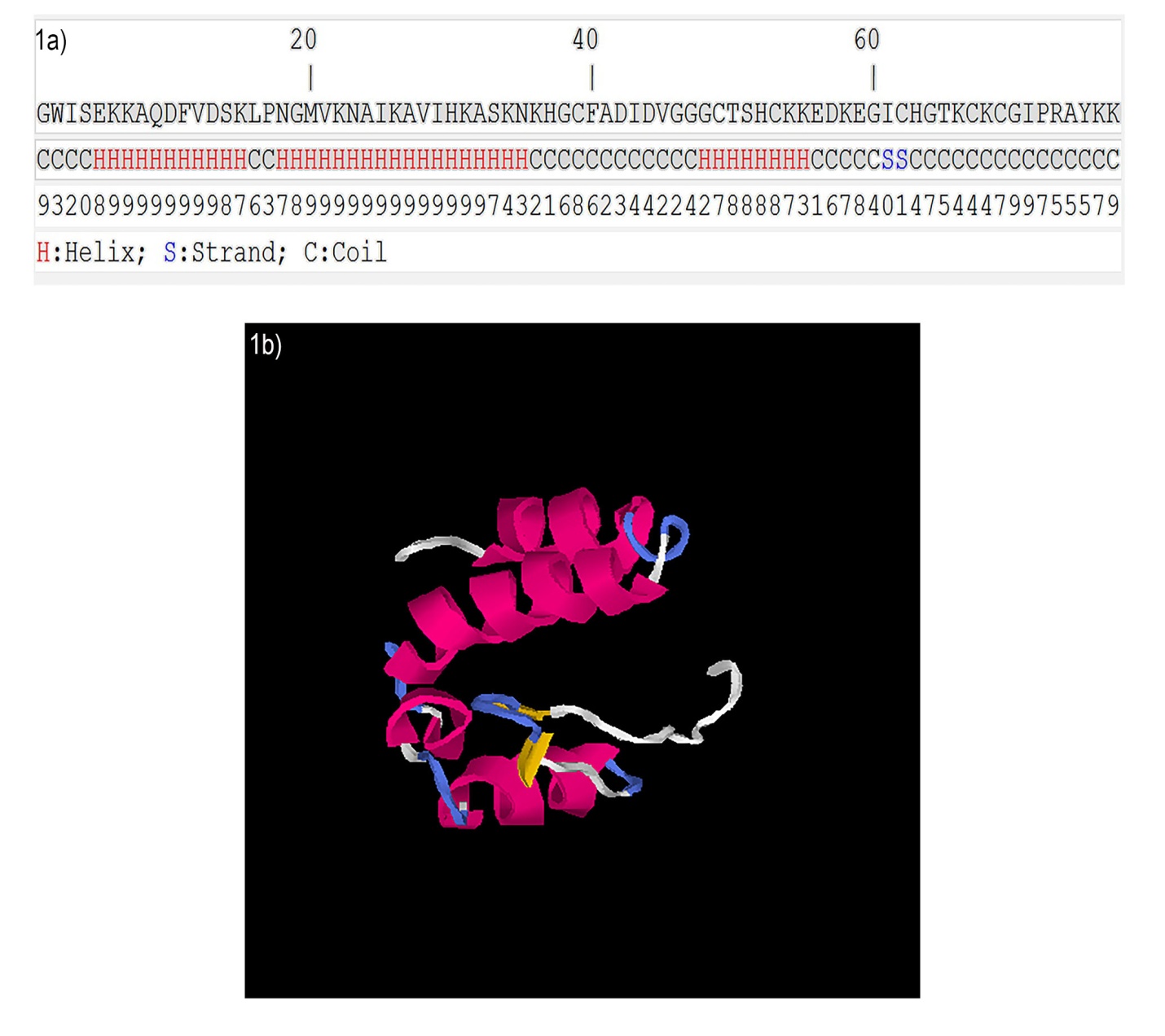


**Additional file 1: Figure S1.** The amino acid sequence and depiction of the three-dimensional structure of scorpine like molecule (SLM). (a) SLM sequence showing predicted domains with helix-coil structure. (b) 3-D structure of SLM as depicted by I-TASSER.
