# Supplementary material for: A paratransgenic strategy to block transmission of Xylella fastidiosa from the glassy-winged sharpshooter Homalodisca vitripennis
Source: BMC Biotechnol. 2018 Aug 22;18:50. doi: 10.1186/s12896-018-0460-z (PMC6104007; doi:10.1186/s12896-018-0460-z)
Supplement: Supplementary file 2 — Figure S2. (a) Assembly and working mechanism of Escherichia coli hemolysin secretion system. HlyB and HlyD form pores in the internal membrane of Gram negative bacteria. These pores join pores formed by TolC in the outer membrane and provide a passage to proteins with the HlyA secretion signal. (b) Depiction of cloning of AMP genes in pEHLYA2-SD plasmid. (DOCX 137 kb) [file 12896_2018_460_MOESM2_ESM.docx]

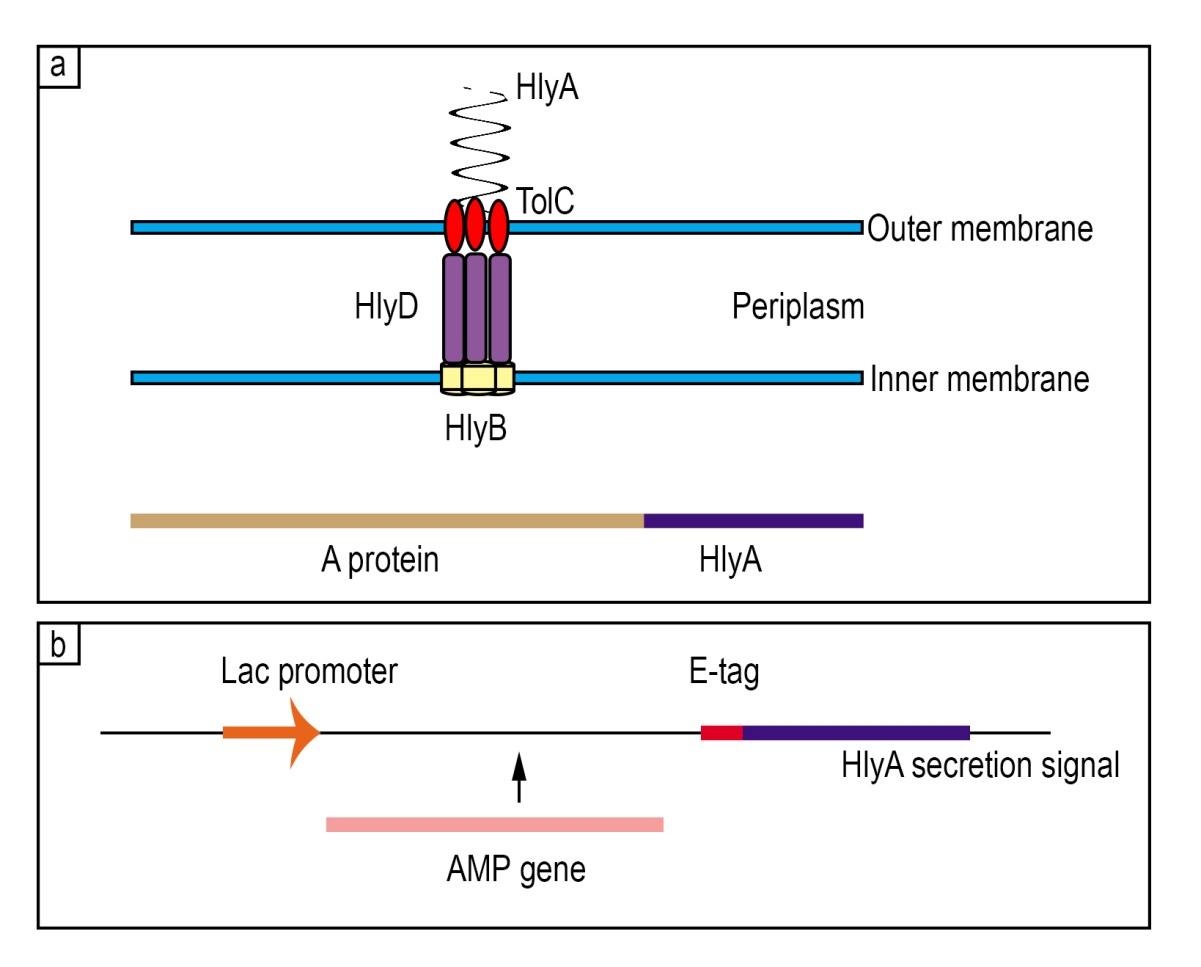


**Additional file 2: Figure S2.** (a) Assembly and working mechanism of *Escherichia coli* hemolysin secretion system. HlyB and HlyD form pores in the internal membrane of Gram negative bacteria. These pores join pores formed by TolC in the outer membrane and provide a passage to proteins with the HlyA secretion signal. (b) Depiction of cloning of AMP genes in pEHLYA2-SD plasmid.
