# Supplementary material for: A paratransgenic strategy to block transmission of Xylella fastidiosa from the glassy-winged sharpshooter Homalodisca vitripennis
Source: BMC Biotechnol. 2018 Aug 22;18:50. doi: 10.1186/s12896-018-0460-z (PMC6104007; doi:10.1186/s12896-018-0460-z)
Supplement: Supplementary file 3 — Figure S3. Confirmation of secretion and accumulation of melittin conjugated to HlyA secretion signal by transformed P. agglomerans lines in spent media as well as within the sharpshooter gut. (a) Spent media were concentrated and were analyzed using an anti-melittin bleed via Western blot. Lane 1: melittin conjugated to HlyA secretion signal; lane 2: synthetic melittin; lane 3: ladder. (b) A sharpshooter homogenate was analyzed using an anti-melittin bleed. Lane 1: ladder; lane 2: sharpshooter fed on P. agglomerans expressing melittin conjugated to HlyA secretion signal; lane 3: sharpshooter fed on wild type P. agglomerans. Two glassy-winged sharpshooters were tested for the presence of melittin using an anti-melittin bleed and both were positive. (DOCX 157 kb) [file 12896_2018_460_MOESM3_ESM.docx]

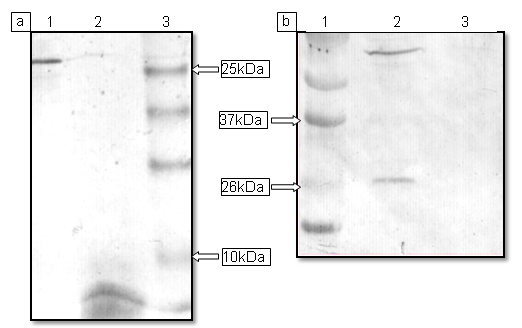


**Additional file 3: Figure S3.** Confirmation of secretion and accumulation of melittin conjugated to HlyA secretion signal by transformed *P. agglomerans* lines in spent media as well as within the sharpshooter gut. (a) Spent media were concentrated and were analyzed using an anti-melittin bleed via Western blot. lane 1: melittin conjugated to HlyA secretion signal; lane 2: synthetic melittin; lane 3: ladder. (b) A sharpshooter homogenate was analyzed using an anti-melittin bleed. Lane 1: ladder; lane 2: sharpshooter fed on *P. agglomerans* expressing melittin conjugated to HlyA secretion signal; lane 3: sharpshooter fed on wild type *P. agglomerans.* Two glassy-winged sharpshooters were tested for the presence of melittin using an anti-melittin bleed and both were positive.
