# Supplementary material for: A paratransgenic strategy to block transmission of Xylella fastidiosa from the glassy-winged sharpshooter Homalodisca vitripennis
Source: BMC Biotechnol. 2018 Aug 22;18:50. doi: 10.1186/s12896-018-0460-z (PMC6104007; doi:10.1186/s12896-018-0460-z)
Supplement: Supplementary file 4 — Figure S4. ELISA to determine detection limit of anti-melittin bleed. Different concentrations of synthetic melittin were prepared and detected via ELISA using anti-melittin bleed. These results are pooled results of two independent experiments. (DOCX 57 kb) [file 12896_2018_460_MOESM4_ESM.docx]

**Additional file 4: Figure S4.** ELISA to determine detection limit of anti-melittin bleed. Different concentrations of synthetic melittin were prepared and detected via ELISA using anti-melittin bleed. These results are pooled results of two independent experiments.
